# Development of a Fast SARS-CoV-2 IgG ELISA, Based on Receptor-Binding Domain, and Its Comparative Evaluation Using Temporally Segregated Samples From RT-PCR Positive Individuals

**DOI:** 10.3389/fmicb.2020.618097

**Published:** 2021-01-20

**Authors:** Farha Mehdi, Souvick Chattopadhyay, Ramachandran Thiruvengadam, Sarla Yadav, Manjit Kumar, Sangita Kumari Sinha, Sandeep Goswami, Pallavi Kshetrapal, Nitya Wadhwa, Uma Chandramouli Natchu, Shailaja Sopory, Bapu Koundinya Desiraju, Anil K. Pandey, Asim Das, Nikhil Verma, Nandini Sharma, Pragya Sharma, Vandita Bhartia, Mudita Gosain, Rakesh Lodha, Urpo Lamminmäki, Tripti Shrivastava, Shinjini Bhatnagar, Gaurav Batra

**Affiliations:** ^1^Translational Health Science and Technology Institute, Faridabad, India; ^2^St. John’s Medical College, St. John’s Research Institute, Bengaluru, India; ^3^ESIC Medical College and Hospital, Faridabad, India; ^4^Maulana Azad Medical College and Lok Nayak Hospital, New Delhi, India; ^5^All India Institute of Medical Sciences, New Delhi, India; ^6^Department of Biochemistry/Biotechnology, University of Turku, Turku, Finland

**Keywords:** receptor binding domain, ELISA, SARS-CoV-2, SARS-CoV-2 IgG antibodies, diagnostics, COVID-19, RBD

## Abstract

SARS-CoV-2 antibody detection assays are crucial for gathering seroepidemiological information and monitoring the sustainability of antibody response against the virus. The SARS-CoV-2 Spike protein’s receptor-binding domain (RBD) is a very specific target for anti-SARS-CoV-2 antibodies detection. Moreover, many neutralizing antibodies are mapped to this domain, linking antibody response to RBD with neutralizing potential. Detection of IgG antibodies, rather than IgM or total antibodies, against RBD is likely to play a larger role in understanding antibody-mediated protection and vaccine response. Here we describe a rapid and stable RBD-based IgG ELISA test obtained through extensive optimization of the assay components and conditions. The test showed a specificity of 99.79% (95% CI: 98.82–99.99%) in a panel of pre-pandemic samples (*n* = 470) from different groups, i.e., pregnancy, fever, HCV, HBV, and autoantibodies positive. Test sensitivity was evaluated using sera from SARS-CoV-2 RT-PCR positive individuals (*n* = 312) and found to be 53.33% (95% CI: 37.87–68.34%), 80.47% (95% CI: 72.53–86.94%), and 88.24% (95% CI: 82.05–92.88%) in panel 1 (days 0–13), panel 2 (days 14–20) and panel 3 (days 21–27), respectively. Higher sensitivity was achieved in symptomatic individuals and reached 92.14% (95% CI: 86.38–96.01%) for panel 3. Our test, with a shorter runtime, showed higher sensitivity than parallelly tested commercial ELISAs for SARS-CoV-2-IgG, i.e., Euroimmun and Zydus, even when equivocal results in the commercial ELISAs were considered positive. None of the tests, which are using different antigens, could detect anti-SARS-CoV-2 IgGs in 10.5% RT-PCR positive individuals by the fourth week, suggesting the lack of IgG response.

## Introduction

SARS-CoV-2, a member of the family Coronaviridae, is the causative agent of coronavirus disease 2019 (COVID-19) CSGICTV (2020). Coronaviruses (CoVs) are a large group of enveloped, single-stranded RNA viruses that predominantly cause respiratory illnesses of varying severity in humans and animals (Chen et al., 2020). CoVs can be broadly classified into four groups: α, β, γ, and δ. SARS-CoV-2 belongs to genera beta coronaviruses and is phylogenetically related to SARS-CoV (Chen et al., 2020). Other human CoVs in this group are MERS-CoV and common-cold causing human-CoVs HKU-1 and OC43 (Chen et al., 2020). SARS-CoV-2 outbreak emerged in late 2019 and was declared a pandemic by the World Health Organization on March 12, 2020 with 21.3 million confirmed cases across the globe as of August 16, 2020 (WHO, 2020). As there is no vaccine or specific treatment available for SARS-CoV-2, the diagnosis of infection is extremely important to control the pandemic (Cheng et al., 2020). Active infections can be diagnosed by detecting viral RNA or viral antigen in the respiratory samples (Cheng et al., 2020). Anti-SARS-CoV-2 antibody detection is not suitable to diagnose the active viral infection but can determine the exposure (prior infection) and is suitable for seroepidemiological studies (Cheng et al., 2020; Theel et al., 2020). Moreover, the presence of IgG may be linked with immunity (Theel et al., 2020). However, for COVID-19, we do not know the exact correlation between the presence of IgG and protection (Theel et al., 2020).

SARS-CoV-2 has four structural proteins; Nucleocapsid, Spike, Envelope, and Membrane (Chen et al., 2020). Among these proteins, antibody response is primarily targeted toward spike and nucleocapsid antigens (Cheng et al., 2020; Gudbjartsson et al., 2020; Theel et al., 2020). Spike is the outermost protein on the virus surface and contains receptor-binding domain (RBD) that binds to the ACE2 receptor on human cells resulting in virus entry in the host cell (Wang et al., 2020). The antibody response against nucleocapsid seems to be earlier compared to spike protein (Burbelo et al., 2020). However, nucleocapsid is very conserved, and there are chances of cross-reactivity with antibodies induced against seasonal CoVs (Tilocca et al., 2020; Van Tol et al., 2020; Yamaoka et al., 2020). Also, the role of anti-nucleocapsid antibodies in protection is likely to be limited and the antibody response to this antigen does not correlate with *in vitro* neutralization (Ni et al., 2020). The full spike protein also contains conserved epitopes particularly in S2 region which may result in cross-reactivity with seasonal CoVs (Ladner et al., 2020). Considering the relatedness of different coronaviruses, RBD is poorly conserved among all and is the most suitable target for the specific detection of antibodies against SARS-CoV-2 infection (Premkumar et al., 2020). Additionally, RBD is the main target of neutralizing antibodies (Ju et al., 2020; Zost et al., 2020a,b). These observations suggest that the anti-RBD antibodies in individuals are likely to be linked with protection (Vabret et al., 2020; Zost et al., 2020a). Antibody detection assays, targeting RBD, may be useful in seroepidemiological studies (good antibody response and specificity), individual risk assessment and determining the sustainability of anti-RBD antibody response.

Detection of IgG antibodies, rather than IgM or total antibodies, against RBD is likely to play a larger role in understanding the antibody-mediated protection, and vaccine studies (Theel et al., 2020).

There are several reports where RBD has been used as the target for the detection of anti-SARS-CoV-2 IgG antibodies. However, none of these reports have shown any systematic optimization of the conditions for the assay (Amanat et al., 2020; Premkumar et al., 2020; Whitman et al., 2020). Moreover, all these processes require fresh antigen coating on immunoassay plate without any stabilization. Stabilization of antigen coated plate and conjugate allows the use of the test kit anytime without preparing the buffer, plate, and conjugate. Moreover, the dry stable plate and the stable conjugate reduce the batch to batch inconsistency as a large batch can be evaluated through a quality control process. The RBD based IgG detection assays reported by others (Amanat et al., 2020; Whitman et al., 2020) are lengthy and take > 4 h which limit the daily throughput.

We have performed an extensive optimization for the SARS-CoV-2 RBD based IgG ELISA to achieve high sensitivity and specificity. The assay development involved a detailed and systematic analysis of assay conditions in terms of types of blockers, sample diluent, incubation temperature and time, wash cycles, antigen concentration, sample volume, etc. The optimized ELISA was converted into a stabilized kit with assay duration of 70 min. The kit was evaluated with a large panel of pre-pandemic negatives (*N* = 470) including interference prone samples and serum samples from SARS-CoV-2 RT-PCR positive individuals (*N* = 312). The performance of RBD IgG ELISA was compared with two commercial IgG ELISAs and found to be superior.

## Materials and Methods

### Materials

96-well flat-bottom MaxiSorp polystyrene plates (Cat No. 442,404) were purchased from Thermo Fisher Scientific Corporation, United States. Non-fat dry Milk (NFDM) was procured from Bio-Rad. Bovine serum albumin (BSA) fraction V was procured from two sources: Sigma-Aldrich and MP Biomedicals. Casein from bovine milk, Trehalose, ProClin-150 and other routine chemicals were procured from Sigma-Aldrich/Merck. HRP-labeled Goat anti-Human IgG Fcγ specific antibody was from Jackson ImmunoResearch. TMB substrate was procured from BD Biosciences. Expi293 cells used for the expression of RBD protein were procured from Thermo Scientific Corporation, United States.

### Recombinant Protein

SARS-CoV-2 Spike protein (GenBank # QHD43416.1) amino acids 330–526 (330PNITNLCPF–HAPATVCG526) corresponding RBD with c-terminal 6x-his tag and N-terminal secretory signals was expressed in Expi293 cells following the manufacturer’s protocol. Briefly, the RBD expression plasmid was transiently transfected into Expi293 cells at a density of 2.8 millions/ml using ExpiFectimine 293 transfection reagent kit. Five days post-transfection, culture supernatant was harvested by centrifugation. The harvested supernatant was loaded onto Ni-NTA column pre-equilibrated with 50 mM Tris pH 7.4 containing 100 mM NaCl at 4°C. The column was washed further with equilibration buffer and protein was eluted with elution buffer containing 500 mM Imidazole. The Ni-NTA purified RBD was further purified via gel filtration chromatography with a Superdex 200 column (GE Healthcare) in PBS (pH 7.4). The RBD obtained after the gel filtration was used for coating of immunoassay wells.

### Clinical Samples

As negative control, pre-pandemic samples (collected before June 2019) from pregnancy cohort [IEC approval no. 1.8.1 (30)], and fever study [IEC decision no. 1.8.1 (60)] were used. In addition to these, pre-pandemic commercial panels from SeraCare, United States containing samples positive for anti-HCV antibodies, HBsAg and autoantibodies were used. Serum samples from SARS-CoV-2 RT-PCR positive individuals were collected from eight clinical sites in the Delhi- National Capital Region, India after ethics clearance from THSTI ethics committee [approval no. 1.8.1 (91)]. Approvals were also obtained from the institutional ethics committees of the participating hospitals where clinical samples and data were collected. The testing by RT-PCR for these participants was done at an approved laboratory as per the National Testing Strategy of India (ICMR, 2020). Pooled negative human serum was procured from SeraCare, United States. Whereas, pooled positive control was prepared in-house from antibody positive serum samples. Details of the clinical samples is provided as Supplementary Table S1.

### Optimization of Assay Conditions

Polystyrene wells from 96-well flat-bottom plates were coated with 50 μl of recombinant RBD protein diluted to 2 μg/ml in phosphate-buffered saline pH 7.4 (PBS) and incubated overnight at 4°C. The coated plates were washed three times with 500 μl per well of PBST (PBS with 0.1% Tween-20) using automatic 96-well plate washer and blocked with 200 μl per well of blocking buffers prepared in PBS. Following blocking solutions were used: (1) 3% NFDM; (2) 1% BSA (Sigma-Aldrich); (3) 1% BSA (MP Biomedicals); (4) 2% casein. The plates with each blocking buffer were incubated either at 37°C or RT (23 ± 2°C) for 1 h. After the incubation, plates were washed once with PBST. 100 μl of diluted (1:50) human sera in different assay buffers were added per well. Following assay buffers, prepared in PBST, were tested: (1) 1% NFDM; (2) 0.5% BSA (Sigma); (3) 0.5% BSA (MP Biomedicals); 4) 1% casein. Plates containing diluted sera were incubated either at 37°C or RT (23 ± 2°C) for 30 or 60 min. Next, the plates were washed thrice with PBST and incubated either at 37°C or RT for 30 min with 50 μl of HRP-labeled Goat anti-Human IgG Fcγ specific antibody diluted to 1:10,000 in respective assay buffer. The plates were washed three times with PBST and incubated with 100 μl per well of TMB at RT for 10 min. The reaction was stopped by the addition of 100 μl of 1M H_2_SO_4_, and the absorbance was measured on a microplate reader at 450 nm with 650 nm as a reference wavelength. The above experiments were performed with 14 pre-pandemic negative and 15 RT-PCR positive samples.

In another set of experiments, the conditions that showed promising results were further evaluated with different wash cycles (3 or 6-wash cycles) after the sample and conjugate incubation steps to assess the effect on assay performance. Here, 53 pre-pandemic negative and 35 RT-PCR positive samples were used. The other assay conditions remained the same as described before.

Further experiments were performed using assay buffer of different pH and detergent concentrations. The assay buffer composition included four different concentration of Tween-20, i.e., 0.1, 0.25, 0.5, and 1% in PBS with either 1% NFDM or 1% Casein. The assay buffers of different pH were also tested where 1% NFDM or 1% Casein were prepared in either PBST pH 7.4 (0.1%Tween-20) or TBST pH 8.0 (50 mM Tris, 150 mM NaCl, and 0.1% Tween-20). Here, sample and conjugate were incubated at RT for 30 min followed by six washes. Twenty-six RT-PCR positive and 21 negative samples were used for these set of experiments. The other assay conditions remained the same as mentioned before.

### Assessment of Antigen Coating Concentration and Serum Dilution

Initial assay optimization was carried out with 2 μg/ml of RBD antigen and 1:50 dilution of serum sample. In further standardization experiments, optimal antigen coating concentration and the serum sample dilution were determined by coating maxisorp wells with 50 μl of RBD protein diluted to 4, 2, 1, and 0.5 μg/ml in PBS and incubated overnight at 4°C. The plates were washed three times with PBST and blocked with 200 μl per well of 3% NFDM in PBST for 1 h at RT (23 ± 2°C). After 1 h, plates were washed once and then incubated with the different dilutions of samples (1:50, 1:100, and 1:200) in assay buffer containing 1% NFDM in PBST for 30 min at RT. Next, plates were washed 6 times with PBST and 50 μl of HRP-labeled Goat anti-Human IgG Fcγ specific diluted to 1:10,000 in assay buffer was added to the assay wells. The plates were incubated for 30 min at RT, followed by washing six times with PBST. Color development and plate reading were done as described in the previous section.

### Conversion of the Optimized Assay to a Stabilized Kit Format

Polystyrene plates were coated overnight with 2 μg/ml of RBD antigen. Plates were washed three times with PBST and incubated with 150 μl per well of blocking cum stabilization buffer (2% Casein, 3% Trehalose, and 0.05% sodium azide in PBS 7.4) for 2 h at RT(23 ± 2°C). After incubation, plates were tapped down gently to remove the blocking buffer and dried overnight. Once the incubation is complete, the individual plates were placed in aluminum pouch with desiccant. The plates were stored at either 4°C or RT (23 ± 2°C).

Stability of HRP-labeled Goat anti-Human IgG Fcγ conjugate was also assessed. For this purpose, the conjugate was diluted to 1:10,000 in two different conjugate diluents. The in-house conjugate diluent contained TBST with 0.1% Proclin-150 and 1% casein. The other diluent was sourced commercially from SurModics (Stabilzyme HRP). The conjugate diluted in different diluents were stored either at 4°C or RT (23 ± 2°C). The stabilized plates and conjugate were compared with the freshly prepared plate and conjugate, respectively, in ELISA method as described previously.

### Performance Evaluation of Developed Kit

The stabilized anti-RBD IgG ELISA kit was evaluated for its performance by testing large sera panels from SARS-CoV-2 RT-PCR positive individuals (*n* = 312) and pre-pandemic negative samples (*n* = 470). The sample details of the evaluation panels are given in Supplementary Table S1. The kit components were allowed to reach room temperature before the assay. 1× wash buffer was prepared from 10× concentrate. Sample diluent was prepared by dissolving 1.5 g NFDM in 50 ml wash buffer, and samples and controls were diluted to 1:50 in the sample diluent. The plates were washed once with 500 μl per well of wash buffer and incubated with 100 μl per well of diluted test sera and controls. After 30 min of incubation at RT (23 ± 2°C), the plates were washed six times with wash buffer. 50 μl of ready to use conjugate was added to the plates. Conjugate was incubated for 30 min at RT, followed by six washes. 100 μl of TMB substrate was added to each well, and the reaction was stopped after 10 min with 100 μl per well of stop solution. The absorbance was measured on a microplate reader at 450 nm with 650 nm as a reference wavelength. The pooled negative control and pooled positive control were run in each plate. The cut-off from each plate was calculated by taking average OD of triplicate of negative control and addition of 0.2 to this value. Signal to cut-off ratio (S/Co) was calculated as the ratio of OD value from test sample to cut-off value.

### Comparison With Commercial ELISAs

The two commercial ELISA kits used for comparative evaluation were Anti-SARS-Cov-2 IgG antibody detection ELISA (Covid Kavach IgG) from Zydus diagnostics, India and Anti-SARS-CoV-2 ELISA (IgG) from Euroimmun, Germany.

In the Zydus ELISA the wells are coated with inactivated SARS-CoV-2. Whereas, in Euroimmun ELISA wells are coated with S1 protein of SARS-CoV-2. The assays were performed according to the manufacturers’ instructions. For Zydus IgG ELISA, a sample is considered IgG positive if the O.D. value is greater than the cut-off value and P/N ratio (Ratio of O.D. value of test sample to the average O.D. value of the negative control) is more than 1.5. If only one criterion is met, sample is considered negative. If the sample OD falls between the 10% ± ranges of the cut-off, it is considered indeterminate.

For the Euroimmun ELISA a tested sample is considered positive if the ratio (ratio of O.D. of sample to the O.D. of the calibrator) is greater than or equal to 1.1, borderline if ratio is in between 0.8 and 1.1 and negative if ratio is less than 0.8.

### Statistical Analysis

Three serum panels, derived from 312 RT-PCR positive individuals, were used to assess the sensitivity of the IgG ELISAs. Panel 1, 2, and 3 contained serum samples collected between days 0–13, 14–20, and 21–27, respectively, from the onset of symptoms or RT-PCR positivity. For the serum from asymptomatic individuals, duration at the time of blood collection was calculated from the date of RT-PCR testing. For symptomatic individuals, duration at the time of blood collection was calculated from the date of RT-PCR testing or date of onset of symptoms, whichever was the earlier. The specificity of the RBD IgG ELISA was assessed by running 470 serum and plasma samples collected before June 2019. The sample details are provided in Supplementary Table S1. The specificities of the commercial ELISAs (Euroimmun and Zydus) were assessed by running 184 pre-pandemic negative serum samples (common for RBD ELISA).

The statistical analysis was done through GraphPad Prism 8.4.2. Scatter plots were drawn for the continuous variables and compared using unpaired *T*-test. Data with *p* < 0.05 was considered as statistically significant. The difference of the geometric mean value of positive and negative samples obtained from each set of assay condition was considered as a measure of segregation between the two groups. For the comparison of different assay conditions, Receiver Operating Characteristic (ROC) curves were also plotted using GraphPad Prism and Area under the Curve (AUC) was calculated. The agreement between the test kits was calculated using the formula: (positive by both methods + negative by both)/total samples. The prevalence and bias adjusted kappa statistic with 95%CI was calculated using R programming language 3.6.3, version using the package *epiR* (Byrt et al., 1993).

The RBD ELISA and the two commercial ELISAs were also compared by AUC of ROCs, as well as the partial AUCs (pAUCs), which were calculated and plotted in R using the pROC package (Robin et al., 2011). The pAUCs were calculated for 95–100% specificity range and corrected using the method suggested by Mcclish (1989), with the corrected pAUCs having values in the range of 0.5–1.0.

## Results

Our primary objective was to develop a fast, simple yet highly specific and sensitive ELISA to detect anti-SARS-CoV-2 IgG antibodies directed against RBD. We used an indirect antibody immunoassay format where RBD is immobilized on a solid surface. Human antibodies against RBD binds to the immobilized RBD and detected by HRP-conjugated goat anti-human IgG (Fcγ specific). We have conducted extensive optimization experiments listed in Supplementary Figure S1 to identify the most suitable conditions for the assay. Before the start of optimization experiments, a pilot study was conducted to get rough estimate on a suitable antigen coating buffer (PBS), antigen concentration (2 μg/ml), sample dilution factor (1:50), and conjugate dilution (1:10,000) (data not shown).

### Assay Optimization

**FIGURE 1:**
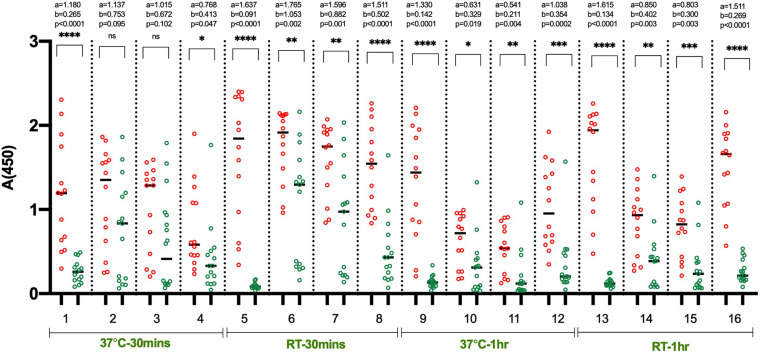
Identification of suitable blocking agent, assay buffer, incubation temperature and time. The scatter plots represent the reactivity of 14 RT-PCR positive and 15 pre-pandemic negative samples with RBD in 16 sets of conditions evaluated. The four blocking agent cum component of assay buffer assessed simultaneously were NFDM (condition 1, 5, 9, 13), BSA-Sigma (condition 2, 6, 10, 14), BSA-MP Biomedicals (condition 3, 7, 11, 15), and Casein (condition 4, 8, 12, 16). The incubation of diluted samples was performed either at 37°C or RT (23 ± 2°C) for 30 or 60 min. Red scatter dots represent the binding profile of positive samples, and green dots represent the binding of negative samples to the coated RBD in each condition. Unpaired *T*-test was used to compare the geometric mean value of absorbance from the positive and negative sets from each assay condition; ‘a’ represents the geometric mean value of absorbance from positive samples, and ‘b’ represents the geometric mean value of absorbance from negative samples for each set of analyzed condition. Absolute *p*-values are also reported for each set of conditions.

**FIGURE 2:**
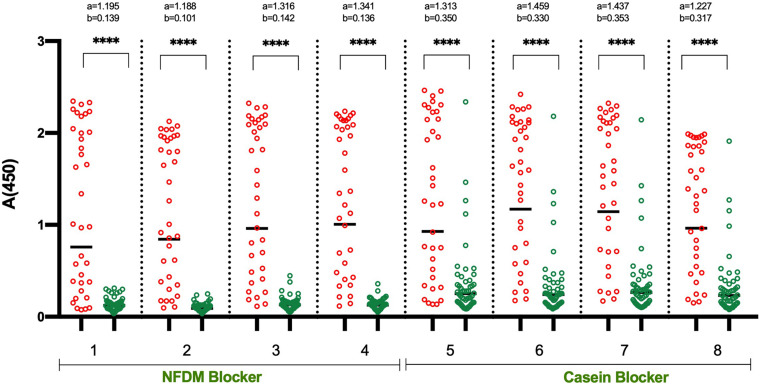
Detailed analysis of shortlisted assay conditions to assess the effect of different wash cycles. The Scatter plots represent the reactivity of 35 RT-PCR positive and 53 pre-pandemic negative samples with RBD in 8 sets of conditions. The two blocking agents cum component of assay buffer were assessed simultaneously: NFDM (condition 1–4) and Casein (condition 5–8). All the incubation steps were performed at RT (23 ± 2°C) with sample incubation time of 30 min (condition 1, 2, 5, and 6) or 60 min (condition 3, 4, 7, and 8). The plates were washed 3-times (condition 1, 3, 5, and 7) or 6-times (condition 2, 4, 6, and 8) between the incubation steps. Red and green scatter dots represents the binding profile of positive and negative samples with RBD. Unpaired *T*-test was used to compare the geometric mean value of absorbance from the positive and negative sets from each assay condition; “a” represents the geometric mean value of absorbance from positive samples and “b” represents the geometric mean value of absorbance from negative samples for each set of condition. *p*-values reported as (****) were < 0.0001.

In the initial set of ELISA optimization experiments, the efforts were focused on identifying the suitable blocking agent, sample diluent, incubation time, and temperature to have good segregation in OD values between positive and negative samples (Figure 1). For this, we used 14 RT-PCR positive and 15 pre-pandemic negative sera. We tested four blocking buffers containing different blockers i.e., non-fat dry milk (NFDM), casein, and bovine serum albumin (BSA) from two sources and the corresponding sample diluent. The incubation steps after the addition of diluted samples were performed either at room temperature (RT) or 37°C for 60 or 30 min. The corresponding temperature was used for conjugate incubation, but with a single incubation duration, i.e., 30 min. NFDM based blocker and sample diluent were found to be most suitable in segregating signals of positive from negative samples, followed by casein (Figure 1 and Supplementary Figure S2). Incubation temperature had a marked effect on the segregation of signals. Incubation at RT was consistently better than 37°C regardless of the blocker used as indicated by higher AUC value for assays performed at RT (Supplementary Figure S2). Following the initial results where NFDM and casein were found to be promising candidates for blocker and sample diluent, further screening was performed to see the effect of wash cycles in conjunction with sample incubation duration. All the steps were performed at RT as this was better than 37°C in the previous experiment. A larger number of positives, i.e., 35 RT-PCR positives that were less than 21 days from RT-PCR positivity or onset of symptoms, and negatives, i.e., 53 pre-pandemic negatives including additional challenging negatives, were used for this experiment (Figure 2). NFDM was again found to be better than casein as indicated by higher AUC values (Supplementary Figure S3). Six wash cycles compared to 3 wash cycles, between the incubations, found to give higher AUC value when the incubation was performed for 30 min (Supplementary Figure S3). The ROC curves for all the eight assay conditions are shown in Supplementary Figure S3 with highest area under the curve (0.968) for condition 2 (NFDM, 30 min incubation at RT and 6-washes).

We evaluated the effect of pH and detergent concentrations (0.1, 0.25, 0.5, and 1% tween 20) in the sample diluent. No positive effect was observed when tween-20 concentration increased from 0.1 to 1% in sample diluent, or the pH was changed from 7.4 to 8.0 (data not shown). We have also checked the effect of plate shaking during incubation and found that there was insignificant effect (data not shown). Additionally, we have checked the three dilutions of the conjugate (1:5,000, 1:10,000, and 1:20,000) and found that 1:10,000 remained most optimal (data not shown).

In all the above optimization experiments, 2 μg/ml of antigen for coating and 1:50 dilution of sera was used based on the pilot study. As the immunoassay optimization is an iterative process, we have again checked the appropriateness of the used antigen concentration and sera dilution with the optimized conditions. For this, four concentrations of antigen and three dilutions of sera were tested (Supplementary Figure S4). Samples that were weakly positive for antibodies were used in this experiment. The best segregation of signals was found to be with the initially determined concentration of antigen and sera dilution (2 μg/ml antigen and 1:50 dilution of sera) (Supplementary Figure S4).

### Conversion to Stabilized Kit

**FIGURE 3:**
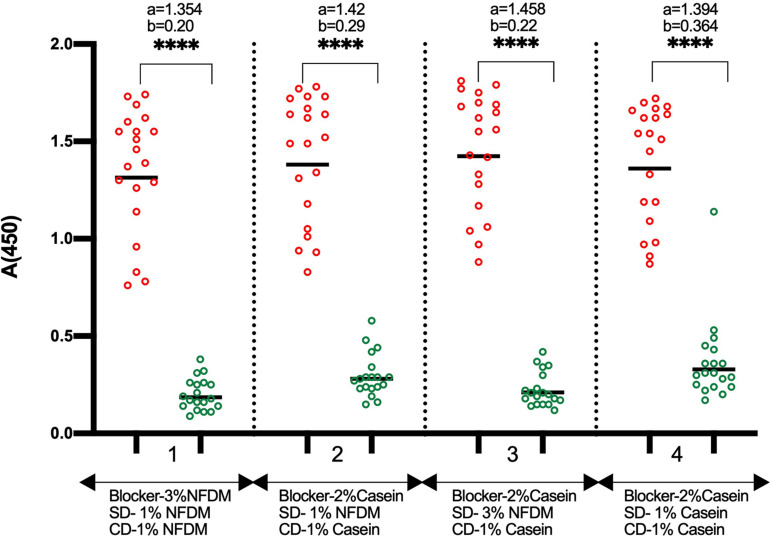
Comparison of NFDM and Casein as a component of blocking and sample diluent. The Scatter plots represent the reactivity of 19 RT-PCR positive and 20 pre-pandemic negative samples with RBD in 4 conditions where NFDM and Casein were evaluated for their effectiveness as a suitable blocker for the RBD coated wells, as sample diluent, and as conjugate diluent. RBD coated wells were blocked with either 3% NFDM (condition 1) or 2% Casein (condition 2–4). Samples were diluted to 1:50 in 1% NFDM (condition 1, 2), 3% NFDM (condition 3) and 1% Casein (condition 4) based sample diluent. The anti-human IgG-HRP conjugate was diluted in either 1% NFDM (condition 1) or 1% Casein (condition 2-4). Unpaired *T*-test was used to compare the geometric mean value of absorbance from the positive and negative sets from each assay condition; “a” represents the geometric mean value of absorbance from positive samples and “b” represents the geometric mean value of absorbance from negative samples for each set of condition. *p*-values reported as (****) were < 0.0001. SD, Sample Diluent; CD, Conjugate Diluent.

Next, we put efforts to convert the assay into a stabilized test format with dry stable plate and ready to use diluted conjugate. We performed an assay where wells were blocked with 2% casein, and freshly prepared NFDM with two concentrations (1 and 3%) was used for sample dilution. The conjugate was diluted in 1% casein solution. This format worked similar to the optimized ELISA having NFDM in all three stages, i.e., blocking, sample dilution, and conjugate dilution (Figure 3). The background was found to be slightly lower with sample diluent containing 3% NFDM. The base buffer for solutions, except coating, was replaced from PBS to TBS because of the compatibility of preservative Proclin-150 with Tris buffer. No difference in the assay performance was observed between the two buffers (data not shown). The RBD coated wells were stabilized with casein-based solution and the dried plates were stored at RT and 4°C. The coated dry plates were found to be stable at both the temperatures (Supplementary Figure S5). The conjugate was diluted in 1% casein-based diluent and stored at 4°C and RT. The conjugate in casein-based diluent remained stable at 4°C (Supplementary Figure S6).

### Cut-Off Determination

Eighty-eight pre-pandemic negative sera and a pooled negative control serum in triplicate were run on three different lots of stable plates to determine the cut-off value. The average OD of 88 negative samples + 3 ^∗^ SD was found to be close to the OD of pooled negative control + 0.2 in the plates from different lots (Supplementary Table S2). In all the further assays, the pooled negative control was run on each plate, and the cut-off was determined by adding 0.2 units to the average O.D. from triplicate of pooled negative control.

### Performance Evaluation of the Developed Kit

**FIGURE 4:**
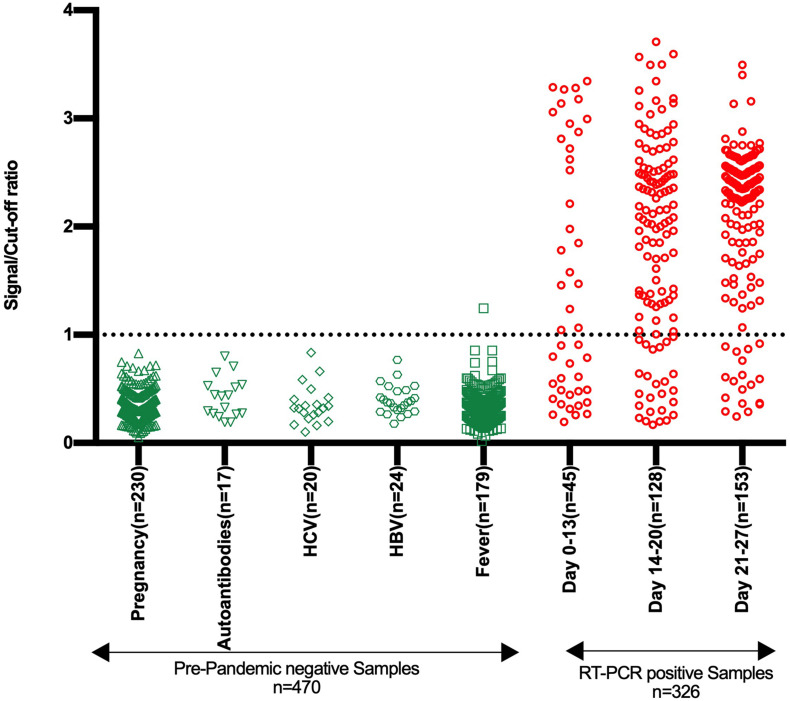
Specificity and sensitivity analysis of stabilized RBD IgG ELISA kit. The Scatter plot represents the reactivity of pre-pandemic negative samples (*n* = 470) and samples from SARS-CoV-2 RT-PCR positive individuals (*n* = 326) in the developed RBD based SARS-CoV-2 IgG ELISA kit. The negative samples belong to five groups, as depicted. The samples from SARS-CoV-2 RT-PCR positive individuals are divided into three groups based on the duration from symptom onset or RT-PCR testing and comprise both symptomatic and asymptomatic category. The reactivity is shown in terms of the signal/cut-off ratio (*Y*-axis). Signal/cut-off ratio ≥ 1 is considered positive, and this point is denoted using a dotted line intersecting the *Y*-axis.

The specificity of the developed RBD based IgG ELISA kit was evaluated on a large panel of pre-pandemic negative samples (*n* = 470) containing different sub-panels (Supplementary Table S1). All the samples, except one from the fever group, were negative in the RBD IgG ELISA (Figure 4 and Table 1). The overall specificity of the test was 99.79% (95% CI: 98.82–99.99%).

**TABLE 1:** Specificity of RBD IgG ELISA kit with pre-pandemic negative panels.

Groups	Samples(*N*)	False positive	Specificity
Pregnancy	230	0	100.00% (98.41–100.00%)
Fever	179	1	99.44% (96.93–99.99%)
Autoimmune	17	0	100.00% (80.49–100.00%)
Anti-HCV	20	0	100.00% (83.16–100.00%)
HBsAG	24	0	100.00% (85.75–100.00%)
**Total**	**470**	**1**	**99.79% (98.82–99.99%)**

**FIGURE 5:**
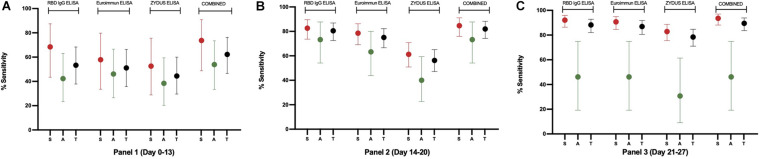
The sensitivity plot of each assay on samples from RT-PCR confirmed individuals. Percent sensitivity is plotted with 95% CI for Symptomatic (S, red), Asymptomatic (A, green), and Total (T, black) samples derived from 31, 128, and 153 RT-PCR positive individuals in Panel 1, Panel 2, and Panel 3, respectively. For this plot, samples that scored equivocal in Euroimmun and Zydus ELISA were considered positive for the sensitivity analysis. Samples found positive in any of the three ELISAs were included in the combined results.

The test sensitivity was determined using three panels containing sera from SARS-CoV-2 RT-PCR positive individuals (*n* = 312). Panels 1, 2, and 3 contained serum samples collected between days 0–13, 14–20, and 21–27, respectively, from the onset of symptoms or RT-PCR positivity. The sensitivity of 53.33% (95% CI: 37.87–68.34%), 80.47% (95% CI: 72.53–86.94%), and 88.24% (95% CI: 82.05–92.88%) were obtained with panel 1, panel 2, and panel 3, respectively (Figure 5 and Supplementary Tables S3–S5). On further categorization of samples based on symptoms, higher sensitivity of 68.42% (95% CI: 43.45–87.42%), 82.65% (95% CI: 73.69–89.56%), and 92.14% (95% CI: 86.38–96.01%) were obtained with Panel 1, Panel 2, and Panel 3, respectively for symptomatic individuals (Figure 5 and Supplementary Tables S3–S5).

### Comparison With Commercial IgG ELISAs

To compare the performance of developed RBD IgG ELISA with the commercial ELISAs, three RT-PCR positive panels were run on Euroimmun IgG ELISA and Zydus Kavach IgG ELISA. In the Zydus ELISA, the wells are coated with inactivated SARS-CoV-2. Whereas, in Euroimmun ELISA, wells are coated with S1 protein of SARS-CoV-2. The results are calculated as per the manufacturers’ instructions. The sensitivity of Euroimmun ELISA was 46.67% (95% CI: 31.66–62.13%) for panel 1, and 67.19% (95% CI: 58.33–75.22%) for panel 2 when equivocal results were counted as negative and was 51.11% (95% CI: 35.77–66.30%) for panel 1 and 75.00% (95% CI: 66.58–82.23%) for panel 2 when equivocal results were counted as positive. The sensitivity of Euroimmun ELISA with panel 3 (21–27 days) was 86.93% (95% CI: 80.54–91.83%), and no sample scored equivocal in panel 3 (Supplementary Tables S3–S5, Figure 5, and Supplementary Figure S7).

The sensitivity of Zydus Kavach IgG ELISA was 44.44% (95% CI: 29.64–60.00%) for panel 1, and no sample scored equivocal in this panel. The sensitivity was 52.34% (95% CI: 43.34–61.24%) for panel 2 and 75.16% (95% CI: 67.54–81.79%) for panel 3 when equivocal results were counted as negative, and 56.25% (95% CI: 47.21–65.00%) for panel 2 and 78.43% (95% CI: 71.06–84.66%) panel 3 when equivocal results were counted as positive (Supplementary Tables S3–S5, Figure 5, and Supplementary Figure S7). As with RBD ELISA, the sensitivity of Euroimmun and Zydus ELISA was higher for symptomatic individuals in all the 3 panels (Supplementary Tables S3–S5 and Figure 5). The RBD ELISA was found to be more sensitive compared to Euroimmun and Zydus ELISA even when equivocal samples in the commercial kits were considered as positive (Supplementary Tables S3–S5, Figure 5, and Supplementary Figure S7). The specificity of Zydus ELISA and Euroimmun ELISA was evaluated using 184 pre-pandemic negative serum samples. These 184 samples were part of 470 samples on which RBD ELISA’s specificity was evaluated. All the pre-pandemic samples on Zydus ELISA scored negative. However, in Euroimmun ELISA, 1 sample scored high positive and 1 scored equivocal (Supplementary Figure S7). The RBD ELISA and the two commercial ELISAs were also compared by calculated pAUC for 95–100% specificity range. As expected, the pAUC in this range was highest for RBD ELISA on all the three panels (Supplementary Figure S8).

When evaluated against each other, the agreement between the tests was maximum with panel 3 (days 21–27) (Table 2). Specifically, the agreement between RBD ELISA and Euroimmun assays improved with the duration of illness/from RT-PCR testing and reached the maximum concordance (97% global agreement) in panel 3 (days 21–27). The agreement estimated by prevalence and bias-adjusted kappa statistic (PABAK: 0.95) was near perfect between the two tests for Panel 3. On the other hand, head-to-head comparison of RBD ELISA and Euroimmun against Zydus Kavach demonstrated that the degrees of agreement were consistently lower than that between RBD ELISA and Euroimmun for all the three panels. The global agreement between the pairs of RBD ELISA and Zydus Kavach, and Euroimmun and Zydus Kavach were 86% (PABAK: 0.71) and 84% (PABAK: 0.69), respectively (Table 2).

**TABLE 2:** Agreement analysis between different ELISAs.

Panel 1 (0–13 days) (Equivocal = negative)*				**Panel 1 (0–13 days) (Equivocal = positive)^$^**			
	
	**RBD ELISA**	**Euroimmun**	**Zydus**		**RBD ELISA**	**Euroimmun**	**Zydus**
	
**RBD ELISA**		80%	87%	**RBD ELISA**		84%	87%
**Euroimmun**	0.6 (0.31–0.81)		89%	**Euroimmun**	0.69 (0.41–0.87)		89%
**Zydus**	0.73 (0.46–0.9)	0.78 (0.52–0.93)		**Zydus**	0.73 (0.46–0.9)	0.78 (0.52–0.93)	
	
Panel 2 (14–20 days) (Equivocal = negative)*				**Panel 2 (14–20 days) (Equivocal = positive)^$^**			
	
	**RBD ELISA**	**Euroimmun**	**Zydus**		**RBD ELISA**	**Euroimmun**	**Zydus**
	
**RBD ELISA**		87%	69%	**RBD ELISA**		95%	73%
**Euroimmun**	0.73 (0.59–0.84)		77%	**Euroimmun**	0.89 (0.78–0.96)		77%
**Zydus**	0.38 (0.2–0.53)	0.55 (0.38–0.69)		**Zydus**	0.45 (0.28–0.6)	0.53 (0.37–0.67)	
	
Panel 3 (21–27 days) (Equivocal = negative)*				**Panel 3 (21–27 days) (Equivocal = positive)^$^**			
	
	**RBD ELISA**	**Euroimmun**	**Zydus**		**RBD ELISA**	**Euroimmun**	**Zydus**
	
**RBD ELISA**		97%	86%	**RBD ELISA**		97%	89%
**Euroimmun**	0.95 (0.87–0.99)		84%	**Euroimmun**	0.95 (0.87–0.99)		88%
**Zydus**	0.71 (0.58–0.82)	0.69 (0.55–0.79)		**Zydus**	0.78 (0.66–0.87)	0.75 (0.63–0.85)	

The values above the diagonal are percentage agreement calculated as (positive by both methods + negative by both)/total samples. The values below the diagonal are kappa statistic with 95% CI in brackets. *Samples equivocal in commercial ELISAs were counted as negative. ^$^Samples equivocal in commercial ELISAs were counted as positive.

## Discussion

RBD is a promising target for detecting anti-SARS-CoV-2 antibodies as it has a highly distinct sequence compared to seasonal coronaviruses and induces robust antibody response (Premkumar et al., 2020). RBD-based IgG detection assays are useful not only for serosurveillance purposes but also required to study the sustainability of anti-SARS-CoV-2 IgGs after infection and vaccination as RBD based IgG assays show high concordance with virus neutralization assays (Premkumar et al., 2020).

Most of the reported RBD based IgG immunoassays are only suitable for academic settings as they require freshly prepared components. These assays take > 4 h apart from the preparation of reagents each time (Amanat et al., 2020; Premkumar et al., 2020). Contrary, a stabilized ELISA test can be used any time with more certainty.

We aimed to develop a fast, highly specific, sensitive, and stable ELISA test with ready to use components. Before starting the assay optimization work (Supplementary Figure S1), pilot assays were performed to determine the suitable coating buffer, coating antigen concentration, sample dilution factor, and the appropriate conjugate dilution. The coating antigen concentration and the sample dilution factor were similar to what is reported by Amanat et al. (2020). During the optimization process, we have intentionally used negative samples that gave high background in the pilot study, so with a limited number of samples, optimal conditions can be identified. NFDM, followed by casein, was found to be the most suitable blocking agent and assay buffer component (Figure 1 and Supplementary Figure S2). Surprisingly, the use of BSA, as a blocker and the assay buffer component, consistently gave high background signals from negative samples regardless of the source of BSA and assay conditions (Figure 1 and Supplementary Figure S2). We have used BSA from two different sources as it is known that BSA from different sources and lots may differ in performance (Xiao and Isaacs, 2012). Incubation at RT was better than incubation at 37°C in all the combinations tested as indicated by higher AUC values (Figure 1 and Supplementary Figure S2). Incubation at RT removes the need for a specialized plate incubator unlike protocols reported earlier for RBD based IgG ELISA requiring incubation at 20°C (Amanat et al., 2020; Whitman et al., 2020). Testing with an increased number of samples indicated that an increase in the wash cycles from 3 to 6 improves the segregation of signals between positive and negative samples resulting in higher AUC (Figure 2 and Supplementary Figure S3). Similar AUC values were observed between assays having sample incubation time of 30 and 60 min (Figure 2 and Supplementary Figure S3). Sample incubation for 30 min was chosen for further experiments, as this reduces the overall assay runtime.

As we intended to convert the assay into a stable kit, the use of NFDM as a blocker for making stable dry plates is not an appropriate option because of the particulate nature of the NFDM solution. Additionally, NFDM settles from the solution after storage and does not make an ideal conjugate diluent for the diluted conjugate’s long-term storage. We found that the NFDM is necessary only for the sample dilution, and the casein-based solutions can be used for blocking the wells and conjugate dilution (Figure 3). The use of casein for plate blocking and conjugate dilution and NFDM buffer for sample dilution does not cause any incompatibility as both the casein and NFDM are derived from bovine milk. The RBD coated wells were blocked stabilized with casein-based buffer, tested after 10 days of storage at RT and 4°C, and found to be stable at both the temperatures (Supplementary Figure S5). The diluted conjugate in casein-based diluent was tested after 30 days of storage at 4°C and found to be stable (Supplementary Figure S6). However, reduction in signals was observed with the diluted conjugate after 30 days of storage at RT (Supplementary Figure S6). An increase in casein concentration did not improve the diluted conjugate’s stability at RT (data not shown). Though conjugate stability at 4°C is good enough for most settings, we have studied the stability of diluted conjugate in a commercial conjugate diluent and found that the diluted conjugate was stable even at RT after 30 days of storage. No difference in the signals was observed when the conjugate was prepared in casein-based diluent or the commercial conjugate diluent (Supplementary Figure S6).

A negative control-cum-calibrator was prepared (Supplementary Table S2) to remove batch-to-batch inconsistency, which was run in triplicate apart from pooled positive control in each plate. S/co ratio of ≥ 1 was considered positive. The calibrator’s use removes the need to run a panel of negative samples in each test run to determine the cut-off value.

The final test kit contained RBD coated dry stable plate, ready to use diluted conjugate, 10× wash buffer, a pouch of 1.5 g NFDM that should be dissolved in 50 ml of 1× wash buffer to prepare sample diluent, a positive control and a negative control-cum-calibrator (Supplementary Figure S9).

The final kit was evaluated with large panel (*n* = 470) of negative samples collected before June 2019 and belong to different groups, i.e., pregnancy cohort, fever cohort, HCV, HBV, and autoantibodies positive (Table 1 and Figure 4). Only one sample from the fever cohort scored positive (Supplementary Figure S7). The sample corresponds to a febrile pediatric patient positive for dengue IgM antibodies. There are contradictory reports in the literature on the possible false positivity of positive dengue or Zika samples in the SARS-CoV-2 antibody assay (Faccini-Martínez et al., 2020; Lau et al., 2020a,b; Lustig et al., 2020). Based on *in-silico* analysis, one report suggests some similarities between the part of the HR2 domain of the SARS-CoV-2 spike protein and the flavivirus envelope (Lustig et al., 2020). As the HR2 domain is outside the RBD, this cannot explain the positivity of one dengue positive sample in our RBD assay. No sequence similarity was found between SARS-CoV-2 RBD and any sequence from the flaviviruses proteome in our search. We believe that the false positivity observed by us is not due to the cross-reactivity, but because of the non-specific binding further indicated by the low S/Co ratio (1.24) for this sample. The overall specificity of the test was 99.79% (95% CI: 98.82–99.99%) (Table 1), which makes the assay suitable for sero-surveillance (Farnsworth and Anderson, 2020).

The developed kit’s sensitivity was evaluated by running three panels containing serum samples from 312 SARS-CoV-2 RT-PCR positive individuals. The RBD ELISA demonstrated increase in sensitivity with the duration of illness or from RT-PCR testing (Figure 5 and Supplementary Tables S3–S5). In the absence of a gold standard for sero-positivity, the positive reference is RT-PCR which is imperfect and maybe prone for false positives and negatives. The sensitivity of the assay may be impacted by this imperfection. To overcome this, samples from RT-PCR positive groups were tested with two commercial IgG ELISAs to determine if our assay is less sensitive or the samples that were missed in our RBD ELISA do not have antibodies against SARS-CoV-2.

The comparative analysis (Figure 5 and Supplementary Tables S3–S5) showed that our ELISA is rather more sensitive for all the three panels and have sensitivity advantage of up to 5.47 and 24.22% compared to Euroimmun ELISA and Zydus ELISA, respectively, even when the equivocal samples in the two commercial kits were considered as antibody-positive. The largest difference was seen in the early stage samples (Panel 1 and 2). With panel 3 (days 21–27), RBD ELISA still has 1.3% higher sensitivity compared to Euroimmun ELISA.

The three ELISA tests were also compared by plotting the ROC curves and partial ROC curves with the partial area under curve values calculated between 95 and 100% specificity range. The pAUC in the high specificity range (95–100%) was compared as the tests should be highly specific to be suitable for sero- surveillance. The RBD ELISA was found to have highest pAUC, on all the three panels, compared to the two commercial ELISAs (Supplementary Figure S8).

In a head-to-head comparison between the three tests, the tests’ agreement increased when equivocal samples in commercial ELISA were considered positive. The overall higher global agreement was observed between RBD ELISA and Euroimmun ELISA (84, 95, and 97% for panels 1, 2, and 3, respectively) (Table 2). Overall, the higher agreement between the three tests was observed in Panel 3 compared to panels 1 and 2, reflecting that kits behave differently for early-stage samples. The same trend of higher agreement of RBD ELISA with Euroimmun was demonstrated by a near perfect prevalence and bias adjusted kappa statistic (Table 2).

When the samples were characterized based on the presence or absence of the symptoms, higher sensitivity was observed with samples from symptomatic individuals in all the three tests with all three RT-PCR positive panels (Figure 5 and Supplementary Tables S3–S5). When samples from symptomatic individuals were used in the analysis, the sensitivity was 92.14% (95% CI: 86.38–96.01%), 90.71% (95% CI: 84.64–94.96%), and 82.86% (95% CI: 75.58–88.70%) for RBD ELISA, Euroimmun ELISA and Zydus ELISA, respectively, with panel 3 (Figure 5 and Supplementary Table S5). From our analysis, it is evident that all the three tests show lower sensitivity for samples from asymptomatic individuals (Figure 5 and Supplementary Tables S3–S5). This is not surprising as poor antibody response in the asymptomatic individuals and thus lower assay sensitivity in this group is well reported in the literature (Long et al., 2020). In a small study from Bangladesh, only 45% of asymptomatic (*n* = 63) individuals seroconverted after 30 days from RT-PCR testing (Shirin et al., 2020). For the sample panels used by us, poorest sensitivity for asymptomatic samples was observed in late stage panel; panel 3 (days 21–27) (Figure 5 and Supplementary Table S5). This may be due the decline in the antibody titer by 4^th^ week as the rapid decline in the antibody titer is reported for asymptomatic individuals (Ibarrondo et al., 2020). However, this is more speculative because of the small number of samples (*n* = 26, 30, and 13, in panels 1, 2, and 3, respectively) from asymptomatic individuals in our study. The combined sensitivity, when the sample is positive by any of the three tests, could reach 89.54% (83.57–93.90%) for total samples and 93.57% (88.15–97.02%) for samples from symptomatic individuals, in panel 3 (days 21–27). None of the tests could detect antibodies in 37.8, 18.0, and 10.5% of the samples in Panel-1 (days 0–13), Panel-2 (days 14–20), and Panel-3 (days 21–27) (Supplementary Table S6), respectively, suggesting the absence or undetectable levels of anti-SARS-CoV-2 IgG antibodies in these samples. The non-reactivity with 10.5% samples from panel 3 (4^th^ week) is unlikely to be due to the assays’ insensitivity, as the three tests use different antigens, i.e., RBD in our ELISA, S1 subunit in Euroimmun ELISA, and inactivated SARS-CoV-2 in Zydus ELISA, and in the presence of antibodies, at least one test should have shown the reactivity. Literature suggest that not all RT-PCR confirmed individuals mount detectable antibody response even after 4 weeks when tested on multiple antigens and formats (Gudbjartsson et al., 2020; Shirin et al., 2020). A systematic large longitudinal study is required to determine the seroconversion rate in symptomatic and asymptomatic individuals from South Asia. The overall lower sensitivity of the commercial assays in our study compared to the published reports (Marlet et al., 2020; Nicol et al., 2020; Sapkal et al., 2020) is likely to be due to the difference in the test population or the reference. None of the samples, out of 184 pre-pandemic samples, scored positive on Zydus ELISA. However, one pre-pandemic sample scored positive in Euroimmun ELISA with a high ratio of 4.21, and another scored equivocal with a ratio of 1.06 (Supplementary Figure S7). If the equivocal score is considered as positive, the Euroimmun ELISA’s specificity was found to be 98.91% (95% CI: 96.13–99.87%).

The developed RBD IgG ELISA was found to be more sensitive than the other ELISAs tested with the same sample panel and has very high specificity in diverse groups of pre-pandemic samples. High sensitivity and specificity is the result of extensive and systematic optimization of the assay. Contrary to the RBD based IgG ELISAs reported earlier, which require 4–5 h (Amanat et al., 2020; Whitman et al., 2020), the RBD ELISA reported here takes only 70 min of runtime and is stable. Moreover, the runtime for developed RBD IgG ELISA is shorter than the commercial Euroimmun IgG ELISA (120 min) and Zydus IgG ELISA (130 min). The shorter assay duration increases the daily throughput, which is crucial for large serosurvey studies. The developed test may be beneficial not only for sero-surveillance studies but also for the individual risk assessment, evaluation of the sustainability of anti-RBD antibodies after infection or vaccination, and for determining the need for booster dose in the post-vaccine era.

## Data Availability Statement

The original contributions presented in the study are included in the article/Supplementary Material, further inquiries can be directed to the corresponding author/s.

## Ethics Statement

The studies involving human participants were reviewed and approved by the Translational Health Science and Technology Institute. The patients/participants provided their written informed consent to participate in this study.

## Author Contributions

GB conceived the study and monitor the progress. GB and FM wrote the manuscript. FM, SC, SY, MK, and SSi performed the assay development and evaluation experiments. GB, FM, and SC analyzed assay data. TS and SG produced the recombinant protein. UL helped in analysis and gave inputs on manuscript. SB co-conceived the study, designed the clinical cohort, standardized the clinical data, collection and storage of biospecimen, and ensured quality control. RT, NW, and UC designed the clinical cohort, standardized the clinical data and biospecimen collection, managed data repository, phenotyped clinical specimen, and ensured quality control. BK managed data repository and ensured quality control. PK and SSo standardized collection, transport, processing, storage, and distribution of the biospecimen. VB and MG collected clinical data and biospecimen at the clinical sites. AP, AD, NV, NS, and PS assisted in the collection of clinical data and biospecimen from the clinical sites. All authors approved the final manuscript.

## Conflict of Interest

The authors declare that the research was conducted in the absence of any commercial or financial relationships that could be construed as a potential conflict of interest.
