# Supplementary material for: Development of a Fast SARS-CoV-2 IgG ELISA, Based on Receptor-Binding Domain, and Its Comparative Evaluation Using Temporally Segregated Samples From RT-PCR Positive Individuals
Source: Front Microbiol. 2021 Jan 20;11:618097. doi: 10.3389/fmicb.2020.618097 (PMC7854536; doi:10.3389/fmicb.2020.618097)
Supplement: Supplementary file 1 [file Data_Sheet_1.PDF]

Supplementary Information

Development of a fast SARS-CoV-2 IgG ELISA, based on receptor-binding domain, and its comparative evaluation using temporally segregated samples from RT-PCR positive individuals

Farha Mehdi[1], Souvick Chattopadhyay[1], Ramachandran Thiruvengadam[1], Sarla Yadav[1], Manjit Kumar[1], Sangita Kumari Sinha[1], Sandeep Goswami[1], Pallavi Kshetrapal[1], Nitya Wadhwa[1], Uma Chandramouli Natchu[1,2], Shailaja Sopory[1], Bapu Koundinya Desiraju[1], Anil K Pandey[3], Asim Das[3], Nikhil Verma[3], Nandini Sharma[4], Pragya Sharma[4], Vandita Bhartia[1], Mudita Gosain[1], Rakesh Lodha[5], Urpo Lamminmäki[6], Tripti Shrivastava[1], Shinjini Bhatnagar[1*], and Gaurav Batra[1*], for DBT India Consortium for COVID-19 Research.

[1]*Translational Health Science and Technology Institute, Faridabad, Haryana, India*
[2]*St. John's Research Institute, St John's Medical College, Bengaluru, India*
[3]*ESIC Medical College and Hospital, Faridabad, Haryana, India*
[4] *Maulana Azad Medical College and Lok Nayak Hospital, New Delhi, India*
[5]*All India Institute of Medical Sciences: New Delhi, Delhi, India*
[6]*Department of Biochemistry/Biotechnology, University of Turku, Finland*

[*]Correspondence : Gaurav Batra (gaurav.batra@thsti.res.in)

Table S1. Details of clinical samples used in the study

**Panel specification**	**No. of samples**	**Source**	**Remarks**
Pregnancy Cohort	230	THSTI	Pre-pandemic serum samples derived from healthy pregnant women
Pediatric Febrile illness cohort	179	THSTI	Pre-pandemic serum samples derived from pediatric population with febrile illnesses
Anti-HCV positive	20	SeraCare, USA	AccuSet HCV-performance panel (0810-0204). Pre-pandemic plasma.
HBsAg positive	24	SeraCare, USA	AccuSet HBsAg-performance panel (0805-0340). Pre-pandemic plasma.
Autoantibody positive	17	SeraCare, USA	Pre-pandemic plasma.<br>1 RF Ab <1000 IU/mL; 1 RF Ab 1001-2000 IU/mL; 1 RF Ab 2001-4000 IU/mL; 1 RF Ab 4001-5000 IU/mL; 2 AMA Ab pos; 5 ANA Ab pos; 3 TG Ab pos; and 3 TPO Ab pos.
Serum samples from SARS-CoV-2 RT-PCR positive individuals (Day 0-13)	45	THSTI	Serum samples collected from 31 RT-PCR positive individuals between 0-13 days from onset of symptoms or RT-PCR positivity*
Serum samples from SARS-CoV-2 RT-PCR positive individuals (Day 14-20)	128	THSTI	Serum samples collected from 128 RT-PCR positive individuals between 14-20 days from onset of symptoms or RT-PCR positivity*
Serum samples from SARS-CoV-2 RT-PCR positive individuals (Day 21-27)	153	THSTI	Serum samples collected from 153 RT-PCR positive individuals between 21-27 days from onset of symptoms or RT-PCR positivity *
Pre-pandemic Pooled Human Serum	1	SeraCare, USA	Pooled Human Serum (1830-0002)

* For asymptomatic individuals, days are calculated from the date of RT-PCR testing. For symptomatic individuals, days are calculated from the date of testing or date of onset of symptoms whichever was the earlier.

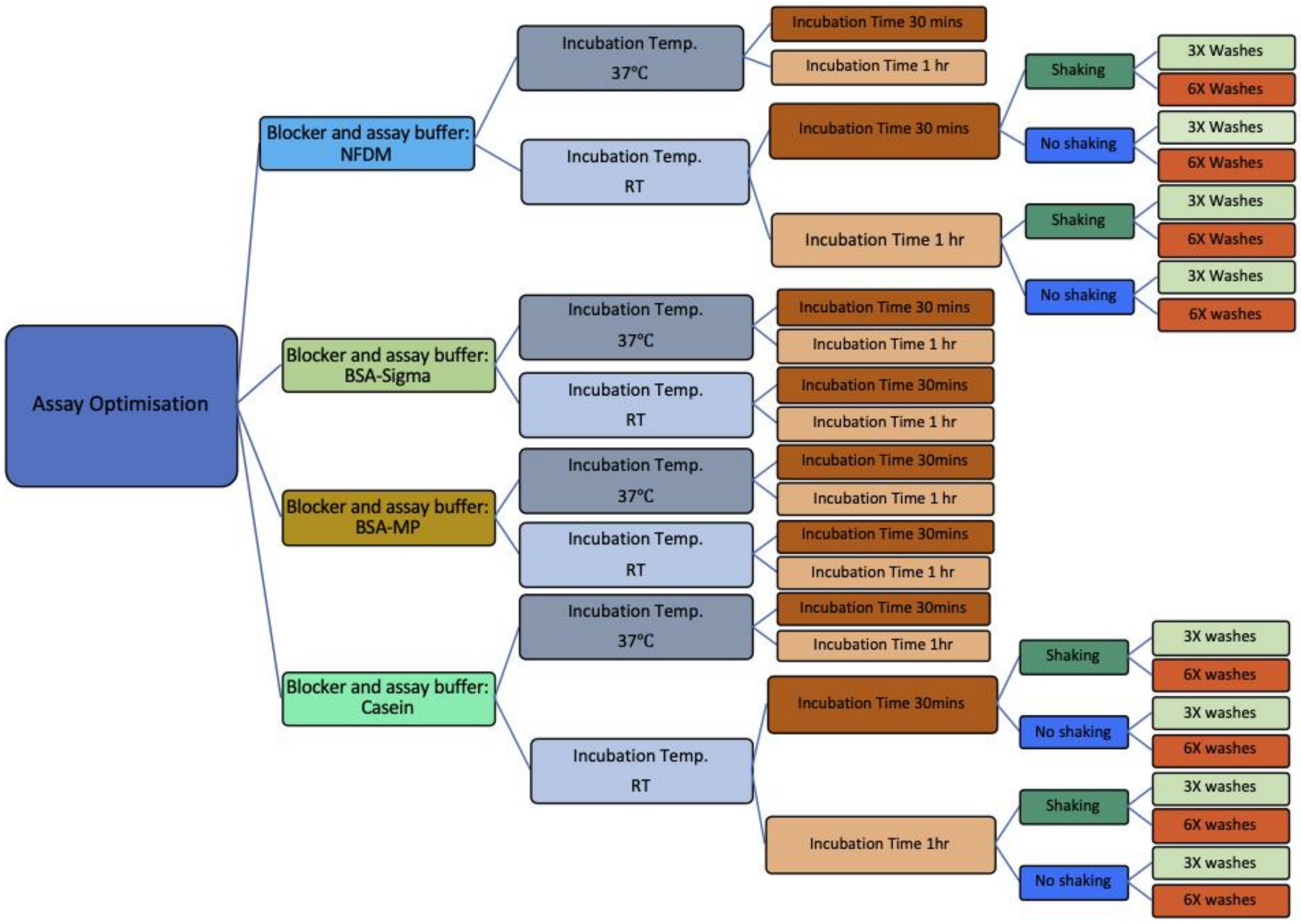

Figure S1. Schematic representation of workflow for the development and optimization of RBD based IgG ELISA. The experiments involved evaluating different blockers as part of blocking and assay diluent along with different incubation time and temperature. The favorable conditions were further evaluated with or without plate shaking with different wash cycles. pH and concentration of detergent in assay diluent were also optimized but not represented in this figure. The different dilutions of the conjugate were also studied but not represented in this figure.

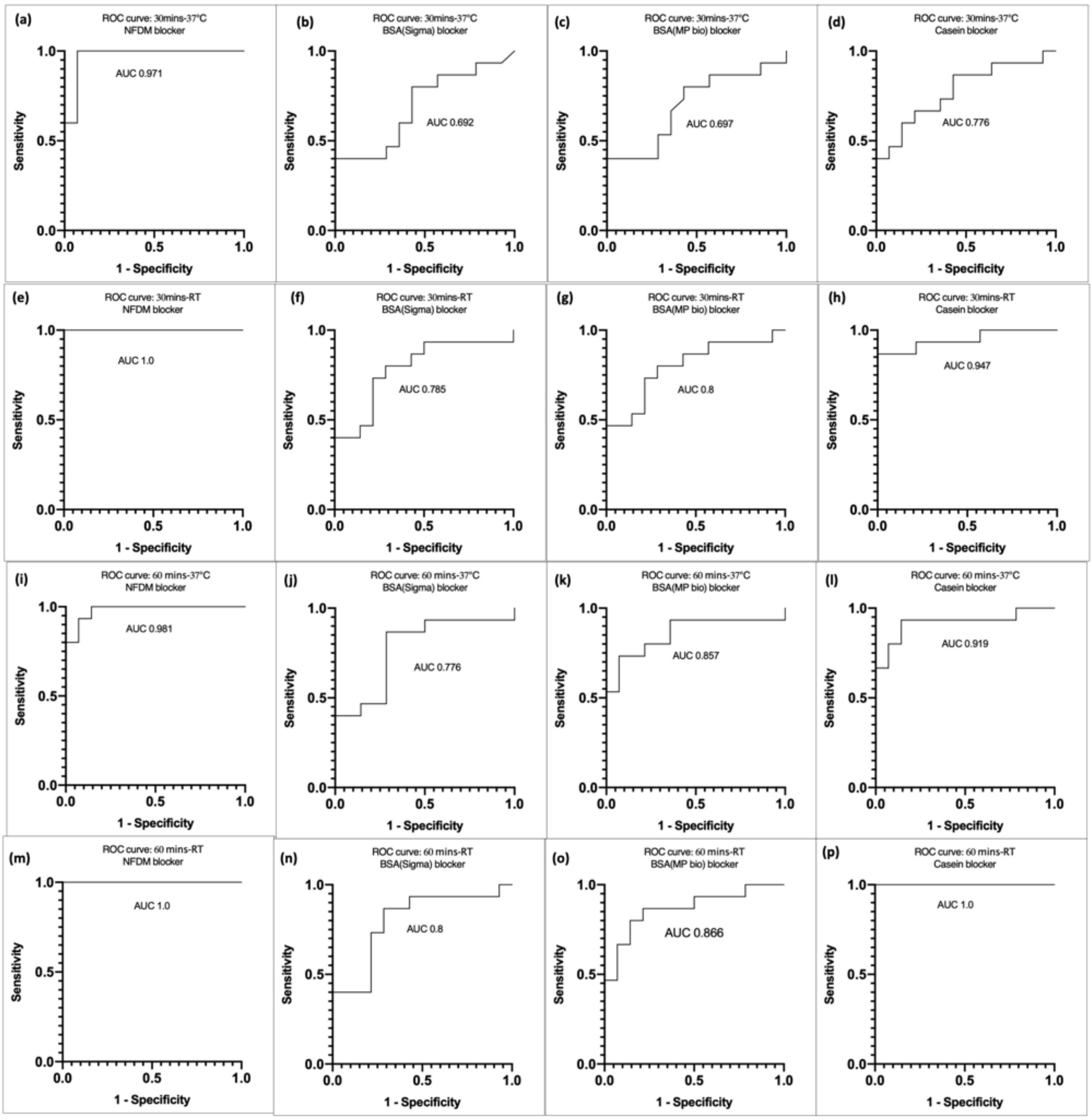

Figure S2. Receiver Operating Characteristics Curves of assay conditions with different blocking agents, assay buffer, incubation temperature, and time. ROC curves were prepared to assess the segregation of signals from 14 RT-PCR positives, and 15 pre-pandemic negative samples in RBD IgG ELISA performed in 16 different conditions. The four blocking agents assessed simultaneously as a part of blocking and assay diluent include NFDM (panel a,e, i, m), BSA-Sigma (panel b, f, j, n), BSA-MP-Biomedicals (panel c, g, k, o) and Casein (panel d, h, l, p). The diluted samples were incubated at 37°C for 30 minutes (a-d), RT(23±2°C) for 30 minutes (e-h), 37°C for 1 hour (i-l), and RT(23±2°C) for 1 hour (m-p). ROC Curves are represented as a plot of 1-specificity on the X-axis and sensitivity on Y-axis with a value for Area Under Curve (AUC).

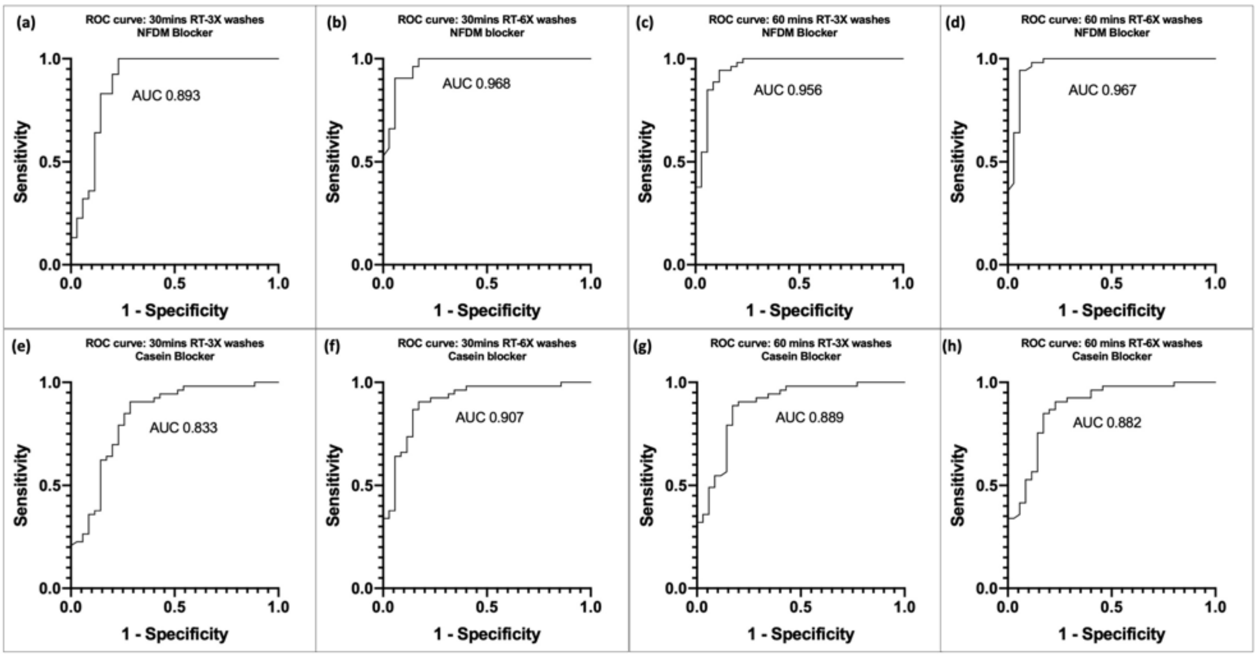

Figure S3. Receiver Operating Characteristics Curves of assay conditions with different blocking agents, assay buffer, incubation time, and wash cycles. ROC curves were prepared to assess the segregation of signals from 35 RT-PCR positives, and 53 pre-pandemic negative samples in RBD IgG ELISA performed in 8 different conditions. The two blocking agents assessed simultaneously as a part of blocking and assay diluent include NFDM (panel a-d) and Casein (panel e-h). The diluted samples were incubated at RT(23±2°C) for 30 minutes (a, b, e, f) or 1 hour (c, d, g, h). The plates were washed 3-times (a, c, e, g) or 6-times (b, d, f, h) between the incubation steps. ROC Curves are represented as a plot of 1-specificity on the X-axis and sensitivity on the Y-axis with a value for Area Under Curve (AUC).

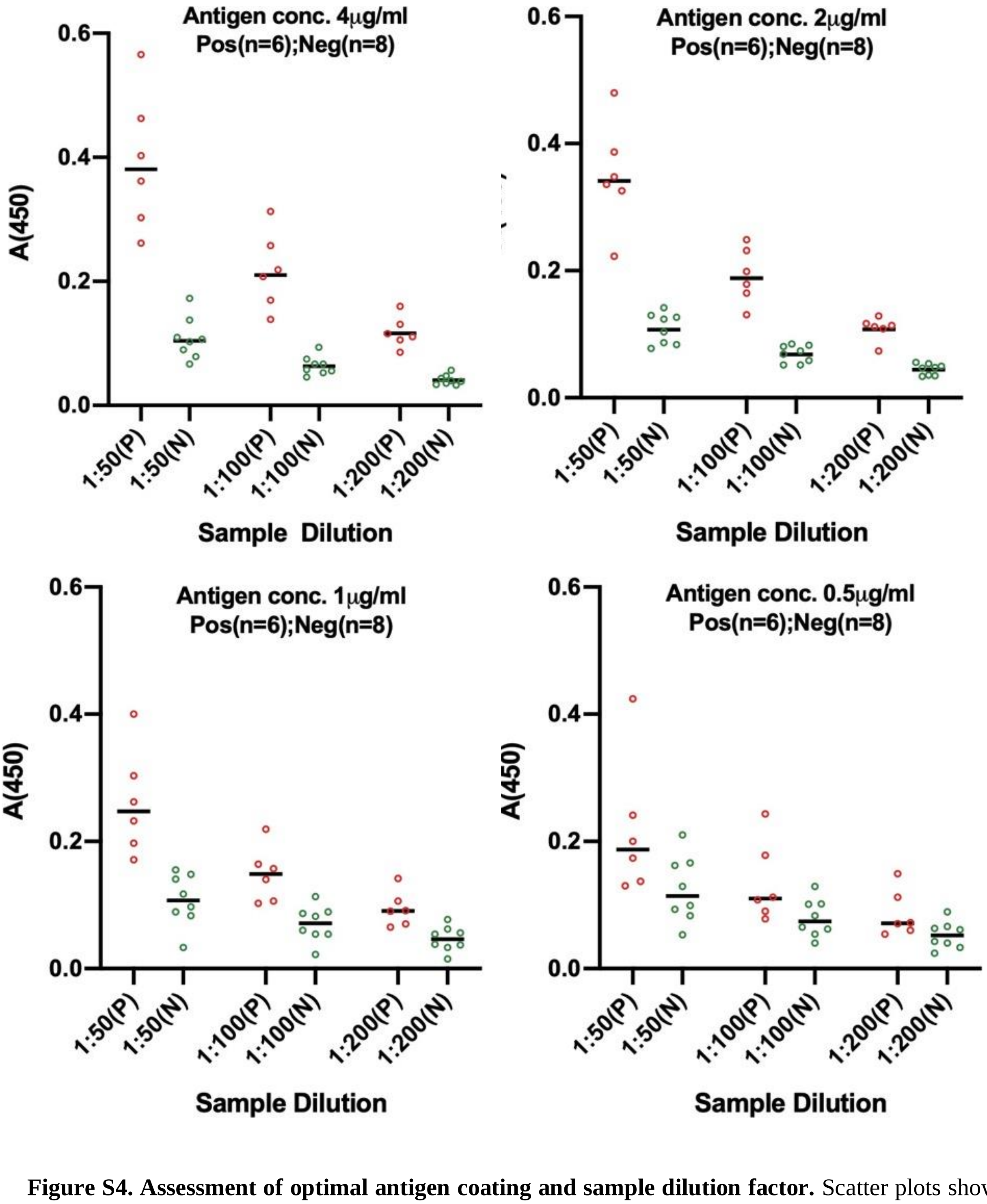

Figure S4. Assessment of optimal antigen coating and sample dilution factor. Scatter plots show the reactivity, of 6 RT-PCR positives and 8 pre-pandemic negative samples, with RBD and were analyzed at four different antigen coating concentrations and three different sample dilution factors. Antigen concentrations were 4µg/ml (A), 2µg/ml (B), 1µg/ml (C) and 0.5µg/ml (D). Each antigen coating concentration was tested with three different dilutions of samples, and signals were measured by taking absorbance at 450nm. In each panel, sample dilution is plotted on X-axis, and the absorbance is plotted on Y-axis. Scatter plots show the absorbance values' geometric mean as a black horizontal line for each positive and negative set of analyzed samples.

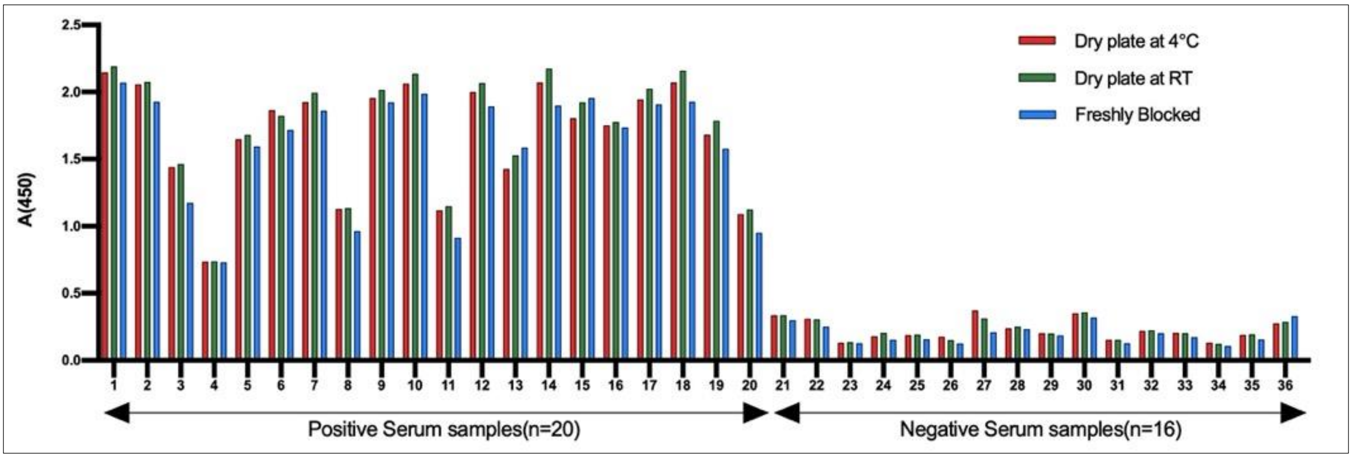

Figure S5. Performance of RBD coated stabilized plate. The bar graph represents the comparative performance of RBD coated freshly blocked plate, and dry stabilized plates post ten days of storage at RT (23±2°C) and 4 °C. The performance was assessed with 20 RT-PCR positive and 16 pre-pandemic negative samples. The X-axis represents the serum samples, and the Y-axis represents the absorbance values. The red bars represent the absorbance from a dry plate stored at 4°C, the green bars represent the absorbance from a dry plate stored at room temperature, and the blue bars represent the sample absorbance from a freshly blocked plate.

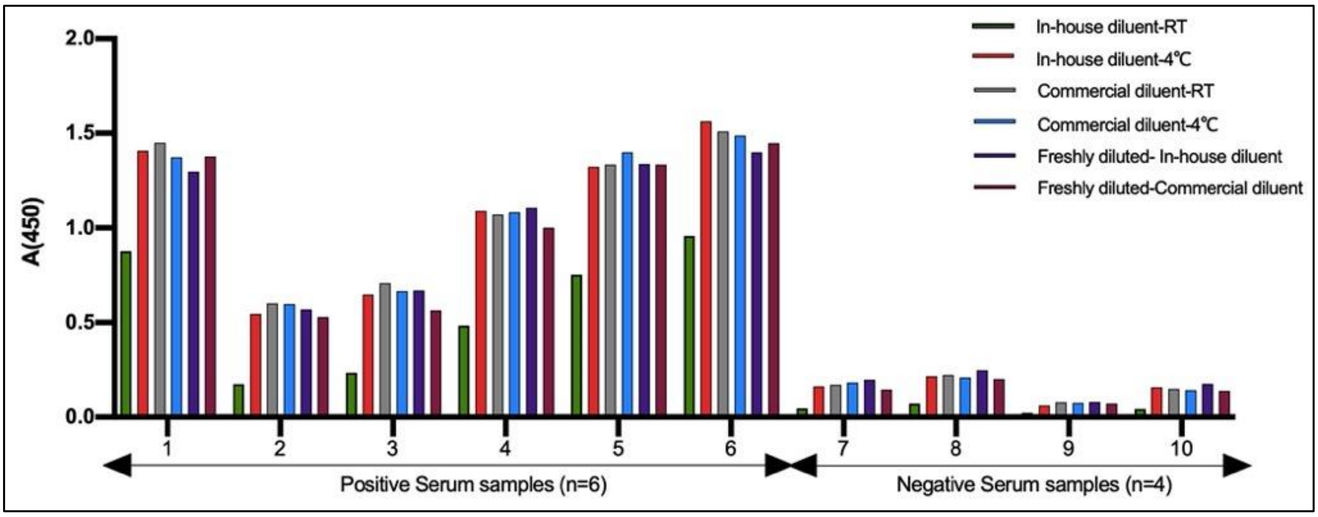

Figure S6. Stability of diluted conjugate at different temperatures. The bar graph represents the comparative performance of diluted anti-human IgG-HRP conjugate stabilized in an in-house diluent and a commercial diluent. The diluted conjugate was stored at 4oC and room temperature for 30 days. The RBD IgG ELISA was performed with six RT-PCR positive and four pre-pandemic negative samples. The X-axis represents the serum samples, and the Y-axis represents the absorbance values.

Table S2. Comparison of average absorbance value of 88 pre-pandemic negative samples with that of pooled negative control serum for the determination of cut-off value

Dry stabilized plate lot	Average O.D. of Neg + 3xSD*	Average OD of pooled negative serum$ + 0.2
Lot 1	0.33	0.40
Lot 2	0.41	0.54
Lot 3	0.5	0.60

*88 pre-pandemic negative sera were used
$pooled negative serum was run in triplicate in each plate

Table S3. Sensitivity of ELISAs for RT-PCR positive panel 1 (Day 0–13)

Category	Days (median)	Samples (N) $	RBD ELISA	Euroimmun (Equivocal = Negative)	Euroimmun (Equivocal as Positive)	Zydus Kavach#	Combined* (positive in any ELISA)
Symptomatic	7	19	68.42% (43.45% to 87.42%)	57.89% (33.50% to 79.75%)	57.89% (33.50% to 79.75%)	52.63% (28.86% to 75.55%)	73.68% (48.80% to 90.85%)
Asymptomatic	5	26	42.31% (23.35% to 63.08%)	38.46% (20.23% to 59.43%)	46.15% (26.59% to 66.63%)	38.46% (20.23% to 59.43%)	53.85% (33.37% to 73.41%)
All	6	45	53.33% (37.87% to 68.34%)	46.67% (31.66% to 62.13%)	51.11% (35.77% to 66.30%)	44.44% (29.64% to 60.00%)	62.22% (46.54% to 76.23%)

$ 45 samples derived from 31 RT-PCR positive individuals
# No sample was equivocal for Zydus ELISA
*Positive in any of the 3 ELISAs. Samples scored equivocal in commercial ELISA were considered as positive

Table S4. Sensitivity of ELISAs for RT-PCR positive panel 2 (day 14-20)								
**Category**	**Days (median)**	**Samples (N)** $	**RBD ELISA**	**Euroimmun (Equivocal = Negative)**	**Euroimmun (Equivocal= Positive)**	**Zydus Kavach (Equivocal =Negative)**	**Zydus Kavach (Equivocal = Positive)**	**Combined* (positive in any ELISA)**
Symptomatic	18	98	82.65% (73.69% to 89.56%)	73.47% (63.59% to 81.88%)	78.57% (69.13% to 86.22%)	59.18% (48.79% to 69.01%)	61.22% (50.85% to 70.90%)	84.69% (76.01% to 91.17%)
Asymptomatic	15.5	30	73.33% (54.11% to 87.72%)	46.67% (28.34% to 65.67%)	63.33% (43.86% to 80.07%)	30.00% (14.73% to 49.40%)	40.00% (22.66% to 59.40%)	73.33% (54.11% to 87.72%)
All	17	128	80.47% (72.53% to 86.94%)	67.19% (58.33% to 75.22%)	75.00% (66.58% to 82.23%)	52.34% (43.34% to 61.24%)	56.25% (47.21% to 65.00%)	82.03% (74.27% to 88.26%)

$ 128 samples derived from 128 RT-PCR Positive individuals
*Positive in any of the 3 ELISAs. Samples scored equivocal in commercial ELISA were considered as positive

Table S5. Sensitivity of ELISAs for RT-PCR positive panel 1 (Day 21-27)							
**Category**	**Days (median)**	**Samples (N)** $	**RBD ELISA**	**Euroimmun** #	**Zydus Kavach (Equivocal = Negative)**	**Zydus Kavach (Equivocal = Positive)**	**Combined* (positive in any ELISA)**
Symptomatic	23	140	92.14% (86.38% to 96.01%)	90.71% (84.64% to 94.96%)	80.00% (72.41% to 86.28%)	82.86% (75.58% to 88.70%)	93.57% (88.15% to 97.02%)
Asymptomatic	21.5	13	46.15% (19.22% to 74.87%)	46.15% (19.22% to 74.87%)	23.08% (5.04% to 53.81%)	30.77% (9.09% to 61.43%)	46.15% (19.22% to 74.87%)
All	23	153	88.24% (82.05% to 92.88%)	86.93% (80.54% to 91.83%)	75.16% (67.54% to 81.79%)	78.43% (71.06% to 84.66%)	89.54% (83.57% to 93.90%)

$ 153 samples derived from 153 RT-PCR positive individuals
# No sample was equivocal for Euroimmun ELISA
*Positive in any of the 3 ELISAs. Samples scored equivocal in commercial ELISA were considered as positive

Table S6. Comparison of 3 ELISAs*

	**Panel 1 (n=45)**	**Panel 2 (n=128)**	**Panel 3 (n=153)**
Negative in all 3 ELISAs	17 (37.8 %)	23 (18.0%)	16 (10.5 %)
Positive in RBD ELISA but negative in Euroimmun and Zydus ELISA	4 (8.9 %)	6 (4.7 %)	1 (0.7 %)
Positive in Euroimmun ELISA but negative in RBD and Zydus ELISA	3 (6.7 %)	0	1 (0.7 %)
Positive in Zydus ELISA but negative in RBD and Euroimmun ELISA	1 (2.2 %)	2 (1.6 %)	1 (0.7 %)

* All the samples (symptomatic and asymptomatic) were used for the calculation. Equivocal samples were considered positive.

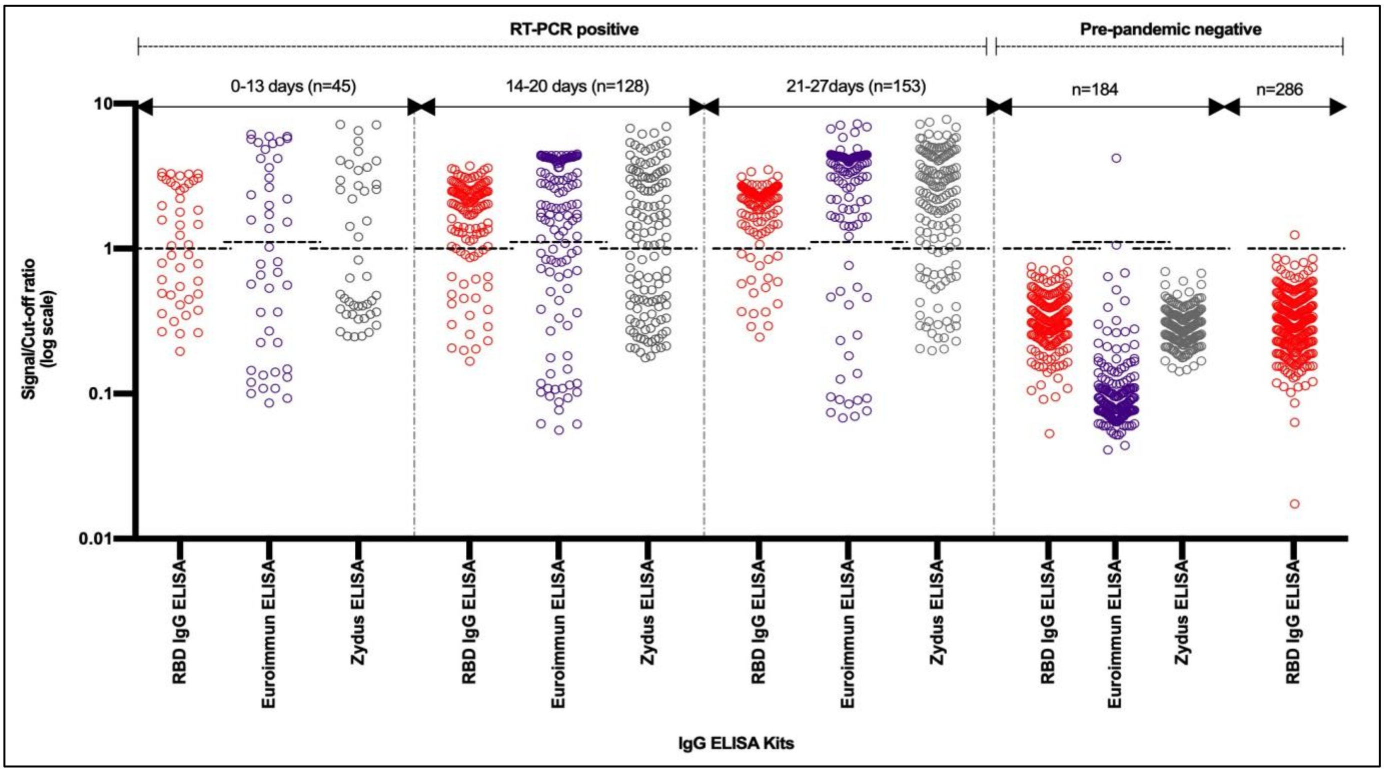

Figure S7. Comparison of SARS-CoV-2 IgG ELISA kits. The Scatter plots represent the reactivity of samples from SARS-CoV-2 RT-PCR positive individuals (n=326) and pre-pandemic negative samples (184 or 470) in the developed RBD based SARS-CoV-2 IgG ELISA kit and the two commercial IgG ELISA kits, i.e., Euroimmun and Zydus Kavach. The samples from SARS-CoV-2 RT-PCR positive individuals are divided into three panels based on the duration from symptom onset or RT-PCR testing and comprise both symptomatic and asymptomatic categories. The specificity of Euroimmun ELISA and Zydus ELISA were determined by running 184 pre-pandemic negative samples. The specificity of the RBD ELISA was determined by running 470 pre-pandemic negative samples (184 common samples used for other ELISAs). The reactivity is shown in terms of the signal/cut-off ratio (Y-axis). The dotted lines intersecting the Y-axis represent the cut-off limit for each ELISA kit, i.e., 1.0 for RBD ELISA and Zydus ELISA, and 1.1 for Euroimmun ELISA.

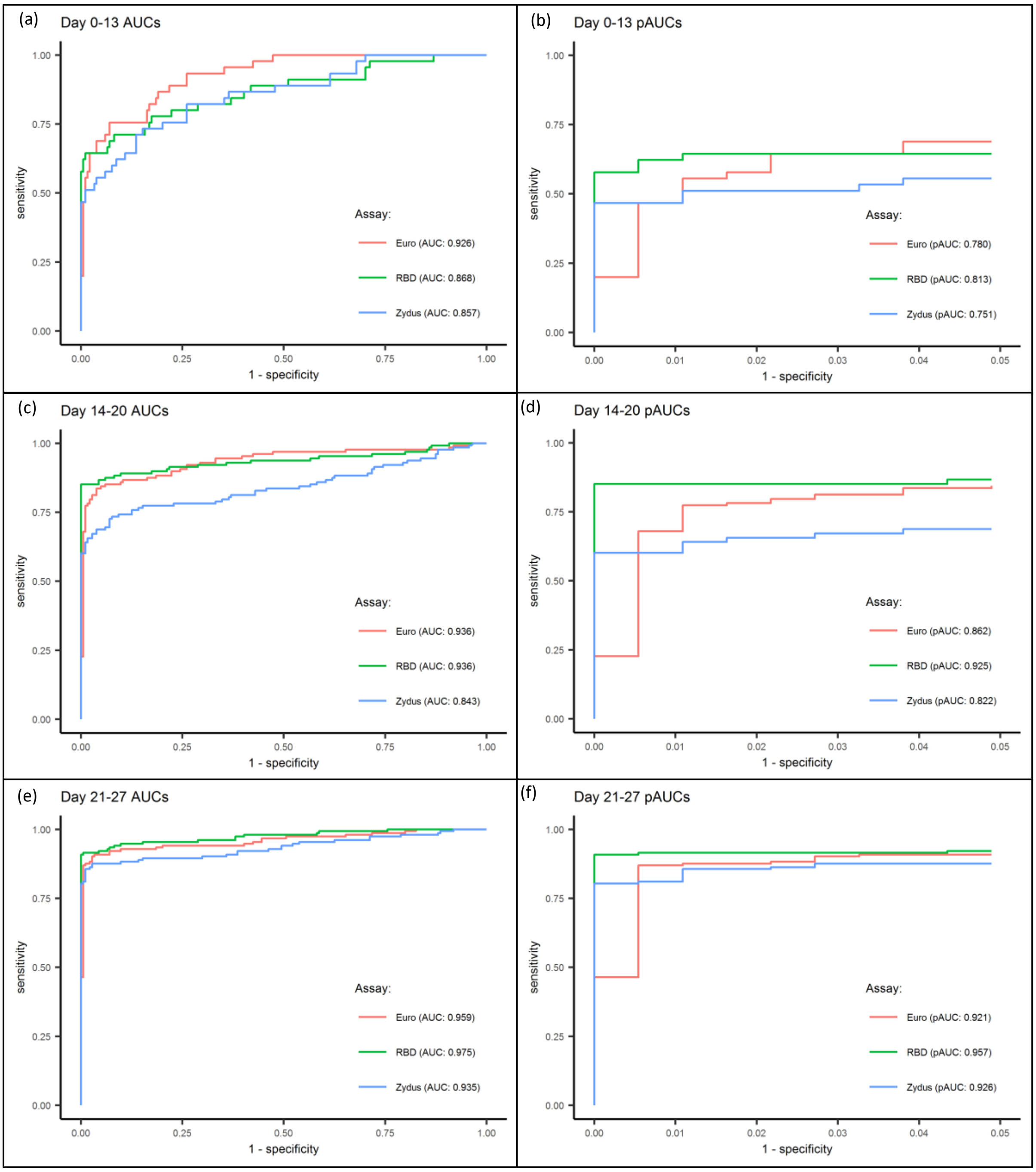

Figure S8: Comparison of SARS-CoV-2 IgG ELISAs using ROC curves. Signal/cut-off ratio obtained from Euroimmun, RBD, and Zydus ELISAs were used to plot the ROC curves. Signal/cut-off ratios of 184 pre-pandemic negative samples were used to plot each ROC curve. Panel a, c, and e show the ROC curves plotted for samples from panel 1 (day 0-13; n=45), panel 2 (day 14-20; n=128), and panel 3 (day 21-27; n=153), respectively, with the area under curve (AUC) mentioned for each test. Panel b, d, and f show the corresponding partial ROC curves for panel 1 (day 0-13), panel 2 (day 14-20) and panel 3 (day 21-27) with the partial area under curve (pAUC) values calculated between 95-100% specificity range as described in the method section. ROC curves are represented as a plot of 1-specificity on the X-axis and sensitivity on the Y-axis.

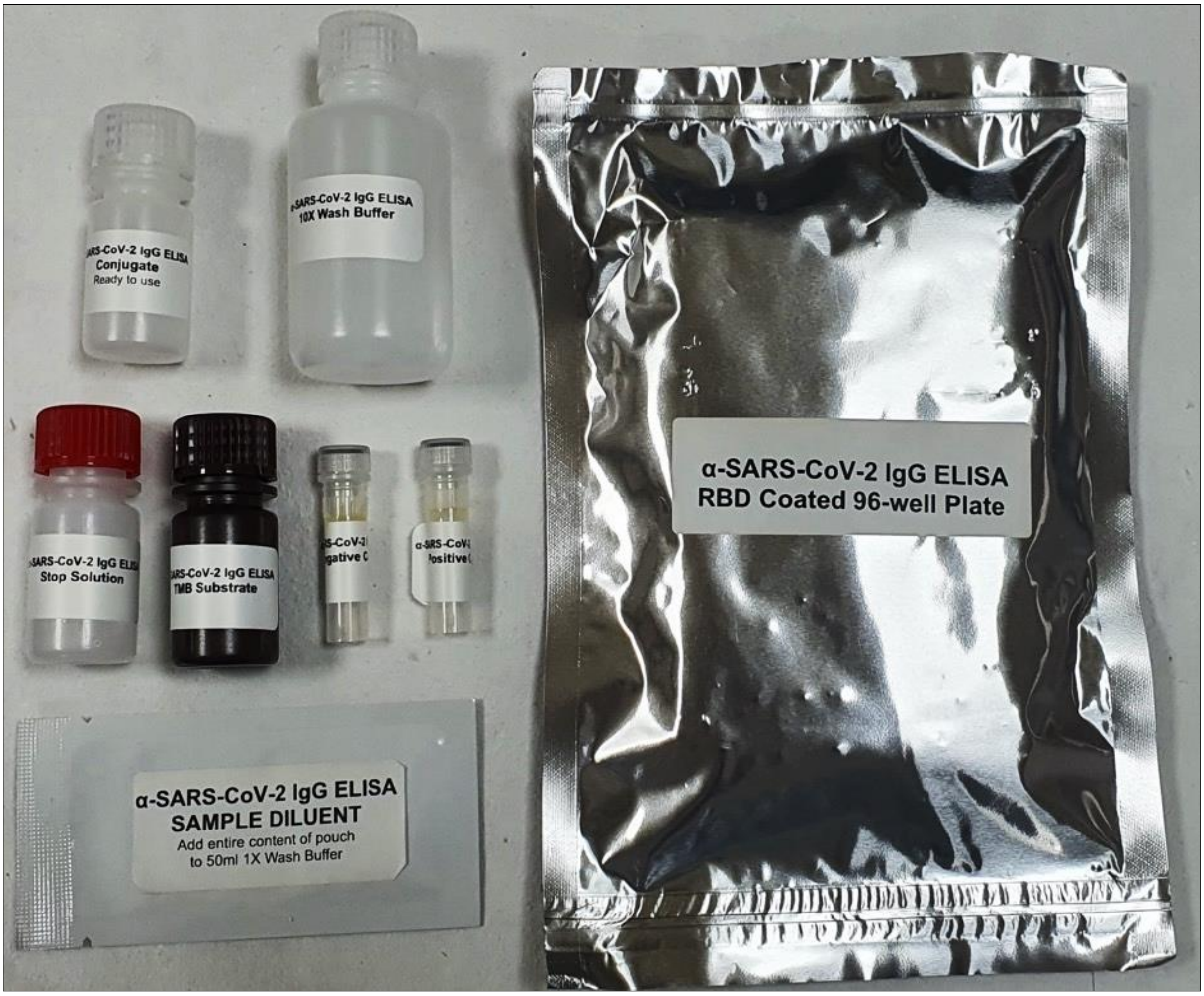

Figure S9: Components of the developed SARS-CoV-2 IgG ELISA kit.
